# Developing precision agriculture using data augmentation framework for automatic identification of castor insect pests

**DOI:** 10.3389/fpls.2023.1101943

**Published:** 2023-02-21

**Authors:** Nitin, Satinder Bal Gupta, RajKumar Yadav, Fatemeh Bovand, Pankaj Kumar Tyagi

**Affiliations:** ^1^ Department of Computer Science and Engineering, Indira Gandhi University, Meerpur, Rewari, Haryana, India; ^2^ Department of Computer Science and Engineering, University Institute of Engineering & Technology, Maharshi Dayanand University, Rohtak, Haryana, India; ^3^ Department of Agronomy and Plant Breeding, Islamic Azad University, Arak, Iran; ^4^ Department of Biotechnology, Noida Institute of Engineering and Technology, Greater Noida, India

**Keywords:** precision agriculture, data augmentation, machine vision, deep learning, insect pests classification, castor

## Abstract

Castor (*Ricinus communis* L.) is an important nonedible industrial crop that produces oil, which is used in the production of medicines, lubricants, and other products. However, the quality and quantity of castor oil are critical factors that can be degraded by various insect pest attacks. The traditional method of identifying the correct category of pests required a significant amount of time and expertise. To solve this issue, automatic insect pest detection methods combined with precision agriculture can help farmers in providing adequate support for sustainable agriculture development. For accurate predictions, the recognition system requires a sufficient amount of data from a real-world situation, which is not always available. In this regard, data augmentation is a popular technique used for data enrichment. The research conducted in this investigation established an insect pest dataset of common castor pests. This paper proposes a hybrid manipulation-based approach for data augmentation to solve the issue of the lack of a suitable dataset for effective vision-based model training. The deep convolutional neural networks VGG16, VGG19, and ResNet50 are then adopted to analyze the effects of the proposed augmentation method. The prediction results show that the proposed method addresses the challenges associated with adequate dataset size and significantly improves overall performance when compared to previous methods.

## Introduction

Castor (*Ricinus communis* L.) cultivation is one of the oldest farming activities in India. India is one of the major castor-producing countries (18.47 lakh tonnes) in the world, followed by Mozambique (0.85 lakh tonnes) and china (0.36 lakh tonnes) in 2020–2021. It contributes a total of 0.15% of the total production of vegetable oil. Castor seeds contain approximately 50% of nonedible vegetable oil. It is a good natural source of hydroxylated and nonhydroxylated fatty acids, which are widely used in chemicals, food, and cosmetics (Salihu et al., 2014). Among the different types of environmental factors faced by castor farming, pests are one of the frequent and important global issues that directly affect agricultural ecosystems and overall crop productivity (Rong et al., 2022). Therefore, to achieve effective and steady plant production, it is necessary to control insect pests and related diseases during the early infestation of the plant. It helps in mitigating the impact of insect pest risks on overall plant production.

Many resistance bioagents and new genetically modified (GM) plants have been developed using recombinant DNA (rDNA) technology to control the effects of insect pests and diseases in castor (Kumar et al., 2013; Kammili et al., 2014; Parthasarathy et al., 2020). However, to reduce the resistance power provided by these advancements, insect pests present mutations in target proteins conferring resistance to common insecticides. Hence, some specific chemicals, such as insecticides and pesticides, are used by agronomists in farming. It helps in the control of insect pest populations, but it also imposes harmful effects if the pesticides used are incorrect or exceed the requirements. Therefore, it is the need of the hour to develop an integrated management technique that combines insect pest detection and a pesticide recommendation system (Karimi and Kamali, 2021). Unfortunately, some farmers used this method for pesticide selection based on their knowledge gained through onsite inspection. It is very time-consuming, and hectic, and requires mastery in the related field. Additionally, due to the expansion and intensification of agriculture as well as the inappropriate knowledge of farmers, it is not possible to conduct farm inspections manually (Yao et al., 2020).

Continuously, advancements in smart devices and image processing techniques help in increasing the interest to adopt modern technologies in agriculture to transform into precision agriculture, which further enables farmers to monitor plant health and potential infestation status. Machine vision technology is novel and successfully applicable in precision agriculture for the automatic pest management system (Dhingra et al., 2022). In this regard, Deguine et al. (2021) developed an integrated pest management (IPM) system to control pests in agricultural farms that took into account multiple considerations related to human health and economic and ecological impacts. These systems require sensors to collect data and send them to processing units for analysis. Moreover, the data collection phase took a long time to complete in order to meet the actual demands, and the shortage of data may be plentiful. Furthermore, a diverse variety of datasets is required to develop an accurate and adaptable strategy for reliable predictions. Usually, to address the issue related to data shortage in machine vision, data augmentation methods are used by researchers to make a classifier more generalized and robust. Thus, Kang (2020) demonstrated a technique using rotation-based data augmentation to train a neural network for wafer map classification. The suggested method provides more consistent results and achieves a higher recognition ratio. In another study, Han et al. (2019) presented image-to-image and image-to-noise data augmentation techniques. The proposed techniques used Generative Adversarial Network (GAN)-based two-step data augmentation process with noise injection in magnetic resonance images and contribute to an overall 3% improvement in performance.

The timely collection of a sufficient quantity of datasets is essential for the proper implementation of the machine vision model (Rosa et al., 2022). In contrast, Dieleman et al. (2015) measured the morphological parameters using deep learning and reflected the limitations of the data augmentation technique. The study showed that as the degree of rotation is increased, label-related data are no longer preserved. To overcome the limitation, Summers and Dinneen (2019) used a noise-based data augmentation technique. However, the proposed technique performed poorly in the case of noiseless data. All of the methods discussed above demonstrate their importance as well as the related limitations of data augmentation. As a result, it still has some room for improvement and requires further research in the agricultural domain for model generalization.

In order to consider these issues, the present study aims to fill a research gap related to previous data augmentation techniques. The authors proposed a castor insect pest classification model based on manipulation-based data augmentation (MBDA) to expand the existing insect pest dataset. This paper mainly contributes:

To recreate an insect pest image dataset for castor crop.
To design a framework for the data augmentation process based on different manipulation-based transformation techniques.
To design a deep learning model using VGG16, VGG19, and ResNet50 for image classification with updated fully connected layers.
To check the impact of data augmentation for evaluation.

Thus, this study aimed to analyze the importance of data augmentation methods in a machine vision system for castor insect pest detection. The proposed system can help related farmers effectively categorize insect pests in real-time and control yield losses. This paper is divided into several sections: the Related works refers to the literature survey, the Proposed data augmentation framework proposes a machine vision-based framework followed by a data augmentation process, and the Materials and method presents the results and discussion. Finally, the Experimental setup and results provides a conclusion to this analysis as well as a future direction.

## Related works

The data augmentation methods are classified into two categories: (1) augmentation of original data (2) and generation of synthetic/artificial data (Mumuni and Mumuni, 2022). The augmentation of original data generally includes pixel transformation, affine transformation, and elastic transformation. While the generation of synthetic/artificial data generally includes principle component augmentation (PCA)-based data generation, GAN-based data generation, and image registration techniques (Nalepa et al., 2019a; Raj et al., 2022). The purpose of this section is to highlight the previous study related to data augmentation and classification techniques. Therefore, this section of the literature is divided into two parts. The first part reported on the literature related to data augmentation, which is widely used for data enrichment, and some of them were compared with the proposed technique, while the second category is related to the machine vision algorithms used for classification.

### Data augmentation

When a dataset is insufficient or unbalanced to train a model, the data augmentation approach is the most appropriate to obtain good prediction results (Xu et al., 2022). The data augmentation method is used in the training process to increase the diversity of data for the machine vision algorithm. It helps to enhance the model’s overall performance and prevent model overfitting. For data augmentation, various types of techniques have been followed by the researcher over the last few decades. It is generally classified as an operation-based manipulation approach, a synthetic data generator approach, and some hybrid techniques are also proposed by different researchers for data augmentation (Raj et al., 2022; Rosa et al., 2022).

#### Operation-based manipulation

Operation-based manipulation is a less complex and important data augmentation approach. It generates new images by performing mathematical manipulation on real-input images. The generic methods in this approach are rotation, flipping, cropping, edge enhancement, noise, and jittering (Shorten and Khoshgoftaar, 2019). Operation-based data augmentation is a label-preserving technique generally illustrated as follows:


(1) $${\{}_{y^{a}=y^{i},}^{x^{a}=f_{m}\left(x^{i}\right),\;}$$

In Eq. (1), transformation is illustrated by the function *f_m_(x)*; where *x^a^* and *x^i^* denote the augmented image and input source image, respectively. Similarly, in the equation, *y^a^* and *y^i^* are the associated labels for images. The equation illustrates that the associated label of the source image is unchanged when the source image undergoes the transformation. In the case of rotation, each pixel of an image is rotated *via* its center. It applies the translation of the object between 0° and 360° angles, and the translation of the object changes the values of coordinates (Kim et al., 2020). Flipping generally involves mirroring pixels across the axis (horizontal or vertical). However, the studies (Hussain et al., 2017; Zhang et al., 2017; Chunling et al., 2022) present a vertical flip-based data augmentation process to capture the vertical reflection for medical imaging. In a study, Kwasigroch et al. (2017) popularized data augmentation strategies for dataset balancing, such as flipping, zooming, translation, and rotation. These techniques are effective and computationally easier to use. In another study, Paschali et al. (2019) proposed affine transformation-based data augmentation that improves the model misclassification problem. It helps to maximize the robustness of the model during the training procedure. This augmentation technique is applied for breast tumors and skin lesion classification problems. However, the use of such techniques in agriculture has not been widely explored. Other than these, kernel and color-space-based transformation are also parts of image manipulation (Tian et al., 2019). The previously mentioned affine transformation enhances the collection since the resulting images are still recognizable and similar. However, the discussed approaches generate irregular and unnatural images. Numerous researchers have considered noise injection to be a useful method that helps to make a model more robust (Zur et al., 2009; Joung et al., 2019). Dong et al. (2022) proposed four annotation strategies aiming to improve disease detection in plants and reduce labeling costs. They include five different types of noise to highlight annotation inconsistency and also explore the extent of the effect on the model’s effectiveness. Krizhevsky et al. (2012) used data augmentation to expand the size of the dataset with a magnitude of 2,048. It used 224 × 224 patches following flips and changes in intensity values in RGB channels for new image creation. This method enhances the size of the dataset and helps to reduce network overfitting during the training. This step helps to improve accuracy and reduces errors by over 1%. Photometric transformation such as color jittering and edge enhancement is also used by the authors for data augmentation (Luke and Geof, 2017).

#### Synthetic data generator approach

New images can be engendered by using conventional image augmentation approaches. However, the final images produced by conventional methods have an equivalent distribution to the input image. These approaches may not be effective whenever the synthetic items lead to data distribution among various subjects. Synthetic data-generating techniques such as GAN, image registration, and PCA generate new synthetic images from the existing images. These procedures may suitable when the synthetic samples have to portray data distribution between many different subjects (Pandian et al., 2019). GANs offer a way to add artificially created samples to the training data. It demonstrated their effectiveness in many areas, such as language and speech processing to image computing (Wang et al., 2017). It has been used in the broad field of agriculture, where artificially generated image samples help to improve the overall accuracy of the D-convolutional neural network (CNN) models, in which the collection of large datasets is not feasible (Tian et al., 2021; Abbas et al., 2021). Image registration techniques have accomplished a remarkable achievement and are also used by various fields for model generalization. In this respect, the researchers (Nalepa et al., 2019b; Abolvardi et al., 2019) proposed registration-based techniques for augmenting multiple medical image datasets. In this technique, two different images of patients are used to generate a new image sample, which smoothly adds features of another image from the first patient image.

#### Other hybrid image data generation approaches

An adequate dataset is required for deep learning-based approaches during the training process to achieve consistent performance. The collection of data is quite expensive and time-consuming in most applications. However, many hybrid approaches have been presented and verified by researchers to tackle this challenge. Meng et al. (2022) proposed a time series data generation method that utilized good quality of plant leaf images. It recognizes three viewpoints for the growth prediction of plants and proposes two new time series data generation algorithms (T-copy-paste and T-mixup). The experiment is performed on KOMATSUNA datasets and achieved effective results. Raj et al. (2022) popularized a new hybrid data augmentation technique based on genetic algorithms. The techniques used crossover between the two images to produce a new image without label preservation. Ozdemir and Sönmez (2022) proposed a D-CNN for the classification of CT scan images of coronavirus disease 2019 (COVID-19) patients. The model used a mixup data augmentation strategy that helps to exploit the additional features and applied them to the publicly available COVID-CT dataset. The strategy achieved significant results and helped in model training as compared to baseline CNN models.

In contrast, the goal of the data augmentation method is to carry out data modifications that will help enhance the model’s generalization. This section of the literature provides a brief overview of the different transformation techniques that are usually adopted by researchers for data augmentation. Despite this, data augmentation in agriculture for insect pest recognition has not been thoroughly investigated and remains a research challenge.

### Machine vision-based approaches for pest detection

This section reports on recent advances in the field of machine vision for detecting insect pests and diseases in agriculture. The related study performed an extensive search on different databases such as Frontiers, Web of Science (WoS), SpringerLink, IEEEXplore, and ScienceDirect with the keywords “pests,” “insects,” “data augmentation,” “deep learning,” and “machine learning.” After initially checking, it is found that a very limited number of publications are related to insect pest detection and classification.

However, Liu et al. (2016) proposed a visual localization pipeline for insect pest classification in paddy field crops. The pipeline first follows a contrast region-based method for pest localization and constructs a database called PestID. This database is used for model training. After training, the outcomes demonstrate that the proposed architecture achieved 95% precision accuracy. Nanni et al. (2020) used two pest dataset collections. One of the used datasets was IP102, which contains 75,000 pest images belonging to 102 different pest classes. The insect pest classification model was tested on five different deep neural network models: GoogleNet, MobileNetV2, AlexNet, ShuffleNet, and DenseNet. Pattnaik et al. (2020) also studied smart agriculture related to pest detection in tomato plants. In this study, the authors collected a tomato insect pest dataset of 859 images from different online sources and classified them into 10 different classes. For testing purposes, 15 different pretrained CNN models were used by the authors. Turkoglu et al. (2022) proposed two models that used deep features of pre-trained CNN to detect diseases in plants. The model used a dataset of 4,447 images with 15 different classes that are collected from different regions of Turkey. The authors used six neural networks for pretraining: AlexNet, ResNet18, GoogleNet, ResNet50, DenseNet201, and ResNet10. The proposed model, PlantDiseaseNet-SEA, based on the idea of sample ensemble averaging with late fusion, achieves an accuracy of 93.6%. PlantDiseaseNet-EF used late fusion to achieve an accuracy of 96.83%. PlantDiseaseNet-MV used the idea of majority voting to select the nearest class and achieve an accuracy of 97.56%. In another study, Cheng et al. (2017b) popularized VGG16 architecture to classify the 10 classes of pests related to different crops. It used Xie’s research pest dataset to train the VGG16 deep neural network and achieved a 95.33% accuracy rate. Furthermore, the model is capable of recognizing the pests in a complex farm background. Huang et al. (2022) established a model based on deep transfer learning to detect eight categories of insect pests. The experiment used a mixed dataset collected from different sources and data augmentation methods (rotation, flipping, and scaling) to enhance the data variability of deep neural networks. Through transfer learning, four deep neural networks—InceptionV3, ResNet, VGG16, and AlexNet—were trained. After the performance analysis, the study found VGG16 achieved the highest accuracy of 94.95% with a standard deviation of 0.44.

A more appropriate model may then be developed that easily differentiates insects that have more analogous traits (Kasinathan and Uyyala, 2021). Different image feature extraction techniques are applied by the authors to evaluate the accuracy. In this study, eight textures, ten shapes, three colors, GIST PCA, and HOG PCA features were used for model training. For pest identification, these feature vector sets are applied to four bases (Naïve Bayes, support vector machine, and K-nearest neighbor) and an ensemble (Bagging, Random Forest, and XGBoost) classifier. In the ensemble classifier, the RF classifier gives a better performance than others with an accuracy of 89.57%, 95.89%, and 91.96%. This research showed that feature vectors have a very great impact on classifier performance.

The machine vision model is still becoming powerful and is successfully used for real-time image recognition. Therefore, the authors discussed various machine vision models that aid in real-time insect detection for castor cultivation.

## Proposed data augmentation framework

After addressing the issue related to the lack of data available for an automated castor insect pest recognition system, the authors then proposed a manipulation-based data augmentation framework. The objective of this research is to increase the variability in imagery data to enhance the learning mechanism in machine vision architecture.

Manipulation-based data transformation techniques are far less complex compared to advanced deep learning methods. It uses various mathematical operations to create new output images without losing the useful features. These methods help in generating different variations of imagery data to improve the randomness in datasets. It is generally classified into five categories (Rosa et al., 2022). In all cases, some of the methods are discussed by the authors due to their consideration of the proposed framework.

### Geometric-based transformation

It is one of the easiest methods that, when used correctly, produces excellent results. It only transforms the coordinates of the input images without affecting their originality. It is the main reason behind the adoption of these techniques with other manipulation-based techniques for data augmentation. In this approach, scaling, rotation, translation, and flipping are the most common geometric transformation techniques that are generally used by many researchers for data augmentation.

### Kernel filter

Kernel filters are n x n matrices that mapped images to produce different effects such as blur, sharpening, and image contrast enhancement. Blurring and sharpening of images with help of filters are the most common techniques used for the data augmentation process. Kang et al. (2017) proposed a patch shuffle regularization data augmentation technique. In this work, the authors used a filter that randomly swaps the adjacent pixels to produce a sharper image. The proposed technique gives a better error ratio than the traditional filter on the CIFAR dataset. The kernel-based techniques are easy to implement and not fully explored by the researchers. Its similarity related to the convolutional neural network (CNN) internal mechanism such as dependencies on multiple different parameters produces some limitations in these techniques for data augmentation (Shorten and Khoshgoftaar, 2019).

**Figure 1:**
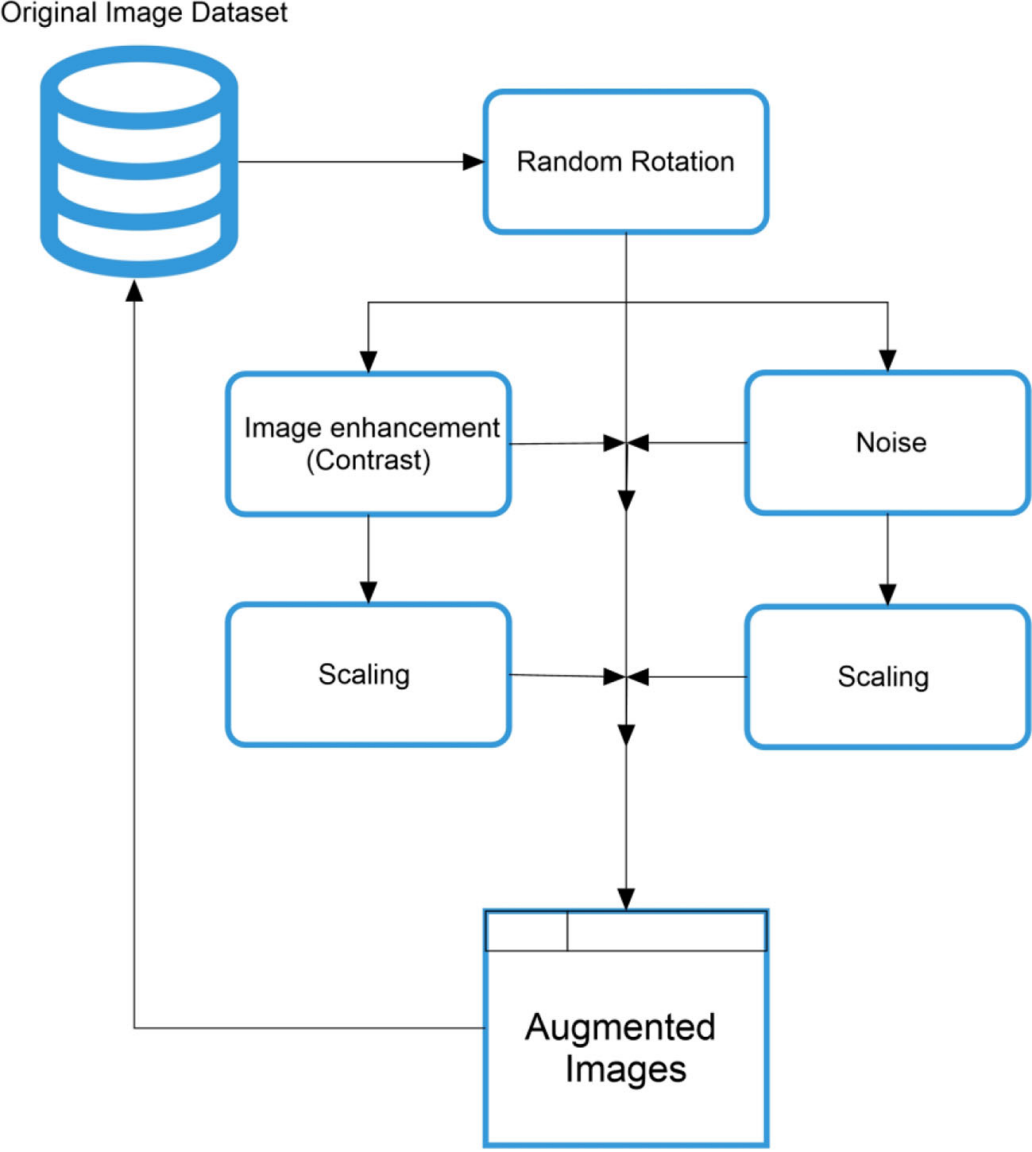
Proposed manipulation-based data augmentation framework.

Despite the utility of the available image dataset, the moderate amount of dataset is an important concern when developing a machine vision model for pest classification. Moreover, the very small differences between the two pest categories—the image mixing, color conversion, and random erasing in pixels—are not suitable for model training. A suitable combination of data augmentation techniques for high-level image transformation is shown in **Figure 1**. Each sample of insect pest images is subjected to multiple operations related to the filter and geometric-based transformation, such as rotation, noise, image enhancement, and scaling. The novelty of the proposed method is to follow a sequential pipeline in which each sample performs multiple operations to increase the randomness in the training dataset.

The proposed framework carefully selected the data augmentation flow. In this case, the first step followed three random rotations for data transformation. In the next step, one set of these rotated images is stored in augmented data, while the other two sets have been subjected to the two distinct transformations. In this procedure, the first transformation in this procedure is image enhancement, and the second is noise addition. After that, the transformed image is also stored in augmented images and moves further for scaling to increase the randomness in the image dataset.

The motivation behind the selection of these manipulation techniques is based on the farmer’s perspective, in which the farmers faced the different forms of images followed by rotation, blurring, and illumination changes.

## Materials and methods

The methodology of the present research is described in this section. At the initial stage, insect pests related to the castor were confirmed, and a dataset of related insect pests was collected from field observation and the Internet.

### Dataset collection and preprocessing

In the present study, original images and some open-source Internet images were used for the experiment. The original images were collected from the fieldwork in a real-time situation. The majority of images were captured using handheld devices such as cell phones (Realme 7, Redmi Note 7 Pro, and others) or cameras (Canon EOS 3000 D). In the collected dataset from the Internet, some images were very similar, even though they were collected from different Internet sources. The visual features contained in these images are almost identical but have some minor differences, such as image brightness or sizes. In these types of situations, only one image of each type was selected for the datasets. Before checking with agricultural experts, we collected a total of 713 images. Thereafter, all collected images were inspected by the agricultural experts who are currently working at the College of Agriculture, Bawal, Haryana, India, and the National Institute of Engineering and Technology, Greater Noida, India. As per the suggestion of related field experts among all images, 341 images from the dataset were eliminated. Finally, the collected dataset contains 372 images and is organized into six different insect pest classes by the experts. In a real-life scenario, the insect pests are different from each other in terms of growth, local names, and species. Two similar insect pests have different local names. Moreover, two similar insect pests have quite similar appearances but seem to be different due to the pest’s life cycle. Therefore, during the process of image acquisition, we depicted the original scientific names to categorize the insect pest classes. The formed dataset was named castor insect pests (CASTIPest), in which each class contained a similar type of image cluster.

**Figure 2:**
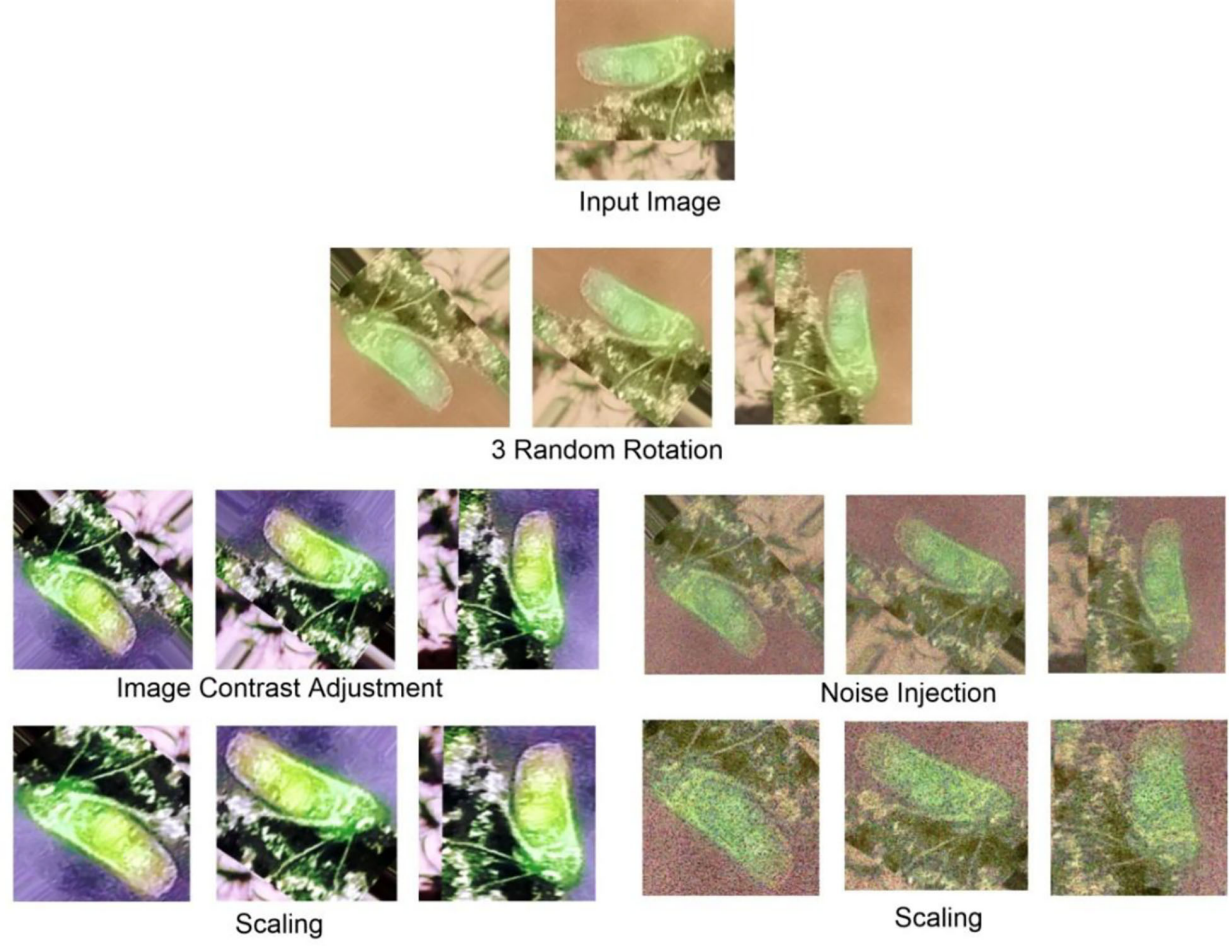
Augmented data of leafhopper image.

**Figure 3:**
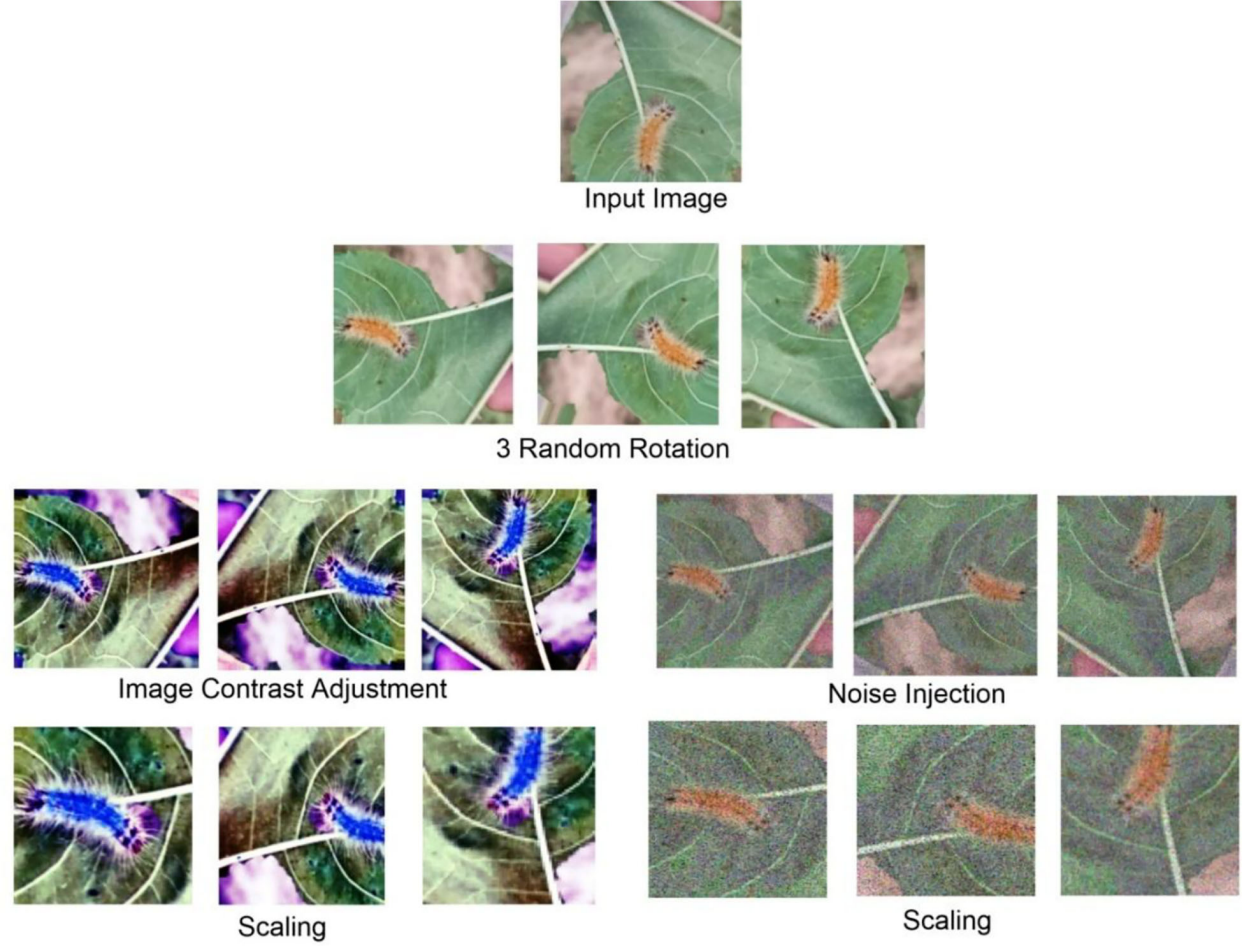
Augmented data of bihar hairy caterpillar image.

It is very difficult for the neural network to train a model accurately in cases of data shortage or imbalance in image classes. It is the main reason behind this study to apply a data augmentation technique for dataset recreation. The number of images produced by the data generation step is given in **Table 1**. Moreover, **Figures 2**, **3** present the image cluster of insect pests that follows the sequence of the augmentation process.

**Table 1:** CASTIPest dataset.

S.N.	Common name of insect pest	Scientific name	Count	Augmented dataset	Abbreviation
**1**	Castor semilooper	*Achoea janata* L.	54	864	AJ
**2**	Bihar hairy caterpillar	*Spilosoma oblique* Wlk.	84	1,332	SO
**3**	Leafhopper	*Empoasca flavescens* (Fabr.)	49	780	EF
**4**	Thrips	*Retithrips syriacus* (Mayet)	69	1,100	RS
**5**	Whitefly	*Trialeurodes ricini* Misra	72	1,136	TR
**6**	Leaf miner	*Liriomyza trifolii* Bergess	44	704	LR

### Implementation of machine vision model

Machine vision-based CNN models are the most common supervised learning models used for classification tasks. Hubel and Wiesel (1959) proposed the first neural network that initially contained one single hidden layer. Due to its limited performance and resources, the CNN model’s training was very difficult during the past decades. Recent advancements in GPU-based processing, high computing speed, and algorithms related to deep learning help efficiently train and established deep learning models. In this case, many CNN models are recently developed by researchers to perform different tasks in multiple fields.

As discussed in the previous section, CNNs give remarkable performance when solving image classification tasks in agricultural fields. A CNN architecture comprises an input layer for the fed input vector, feature extraction layers, one or more fully connected layers, and an output layer. The feature extraction layers generally include convolutional and pooling layers. Convolutional layers are one of the most important layers and serve as a filter to perform automatic feature extraction on images. The pooling layers take the input as a feature vector and help to reduce the sizes of the feature vector without losing the crucial features or information. It also aids in avoiding overfitting issues. The fully connected layers that are responsible for classification are one of the most complex layered structures in any neural network architecture. It frequently calculates the loss with help of some loss function, which allows it to make an exact prediction using an activation function.

In the present study, machine vision models such as VGG16 (Liang et al., 2022), VGG19 (Tetila et al., 2020), and ResNet50 (Malathi and Gopinath, 2021) have been applied to analyze the impact of the data augmentation framework on the classification process. The selected CNN architectures can be trained on augmented and nonaugmented datasets. The motivation behind the selection and architecture of the aforementioned models are discussed below.

#### VGGNet

The architecture of the VGG16 and VGG19 is introduced by Simonyan and Zisserman (2015) to investigate the impact of network deepness on prediction accuracy. Both suggested models showed the best prediction performance on the 2014 ImageNet Challenge. The architecture used 3 × 3 small-sized convolutional filters that help to achieve significant improvement when the depth of weight layers increased from 16 to 19. In VGG16, 13 layers are associated with convolutional, five with pooling, and three with fully connected layers. On the other hand, VGG19 architecture has 16 convolutional, five with pooling, and three with fully connected layers.

#### ResNet

ResNet is also a well-known deep residual network (He et al., 2016). It follows complex network structure than other models that support vanishing gradients and degradation problem solving, which plagued other models. It has used a unique feature named shortcut connection. The shortcut connection jumps from consecutive layers and adds the global features to the output layer. It also reduces training time and maintains degraded performance. In the 2015 ILSVRC competition, it achieved the highest accuracy compared to other models. Another study by Cheng et al. (2017a) demonstrates that the ResNet showed better prediction accuracy than other models in pest classification.

**Figure 4:**
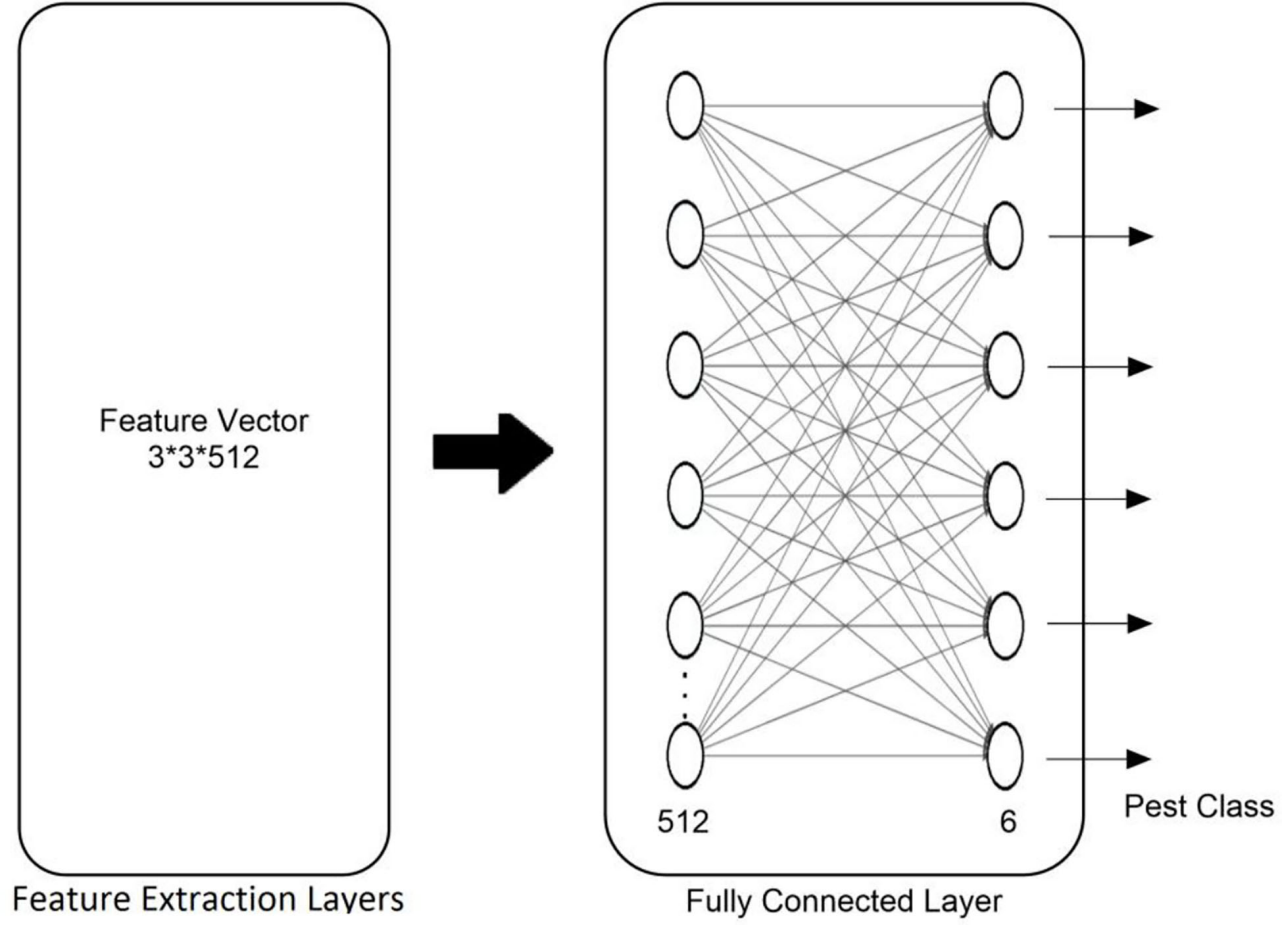
Modified FCL head of the machine vision model.

Image resizing and preprocessing are two of the first steps in preparing images suitable for the model (Liu and Wang, 2021). For VGG16 and VGG19, the authors have chosen to resize the images to 224 × 224 pixels, and ResNet50 to 124 × 124 pixels size. The weights of selected networks have been preserved with fresh training and the help of several hyperparameters. The hyperparameter setting to test the proposed method is shown in **Table 2**. The selected models used batch normalization with a dropout ratio of 0.3 to 0.5, cross-entropy loss, and Adam as an optimizer for the training. The Adam optimizer is used to update the learning parameters and reduce category cross-entropy loss (Kingma and Ba, 2014; Yadav et al., 2021). The selection of the best-suited optimizer to produce optimized results is still an open research issue for researchers. In this regard, Alzahab et al. (2021) published a literature review that reported multiple studies and showed that Adam is an effective optimizer for model optimization. The whole architecture of the machine vision models is divided into two blocks. The first block deals with the feature extraction process and the second block used fully connected layers (dense) for correct prediction. After the feature extraction with help of convolution layers, the flattening of features is carried out by the dense network. The dense head is updated by the authors and consists of two layers. The first layer of the dense network used rectified linear unit (ReLU) as an activation function with a dropout ratio of 0.5 and comprised 512 total nodes. The second layer of the dense network consists of six nodes and softmax as an activation function. The modified head of the machine vision architectures is shown in **Figure 4**.

**Table 2:** Model setup and list of hyperparameters used for training.

Model	Epochs	Optimizer	Dropout range	Batch size	Nontrainable parameters	Trainable parameters	Learning rate	Validation frequency
**VGG16**	100	Adam	0.3–0.5	32	11,520	19,973,702	0.001	1
**VGG19**	100	Adam	0.3–0.5	32	12,032	22,399,302	0.001	1
**ResNet50**	100	Adam	–	32	53,120	27,732,486	0.001	1

The performance of each model on the target dataset is evaluated in terms of accuracy, which is described below:


(2) $$\text{Accuracy}=\frac{\text{Total number of correct predictions}}{\text{Total number of Predictions}}$$

## Experimental setup and results

This section of the study is related to the experimental investigation of the proposed methodology. The entire experiments carried out in this research were done on the Google Colab platform with a Tesla T4 GPU. For the implementation, Python was chosen as a programming language, and different libraries, such as TensorFlow, Keras, OpenCV, etc., were used by the authors for the implementation. In order to analyze the effectiveness of the data augmentation process, two experimental strategies were adopted by the authors. (i) The original image dataset, without any overlapping of samples, is split into train and validation datasets with a ratio of 7:3, to evaluate model baseline performance. (ii) The augmented dataset produced by the benchmarked and proposed approach consists of training. The original image dataset, without any modification, is considered for validation. The dataset distribution of the original dataset and proposed augmented dataset is shown in **Table 1**.

To evaluate the performance of the proposed techniques, some benchmarking methods were selected by the authors as they are widely used in agricultural image augmentation (Pandian et al., 2019). The selection of benchmarking models follows some advanced synthetic data augmentation methods as well as the traditional data augmentation techniques used for the proposed method. The selected state-of-art methods had already been used by the researchers for agricultural image data augmentation. Here, we elaborate on some of the benchmarking methods and compared them with the proposed technique.

### Rotations

It is the most common geometric transformation-based method used for rotate an image on a certain angle value ɵ with the help of the rotation matrix Rt.


.(3) $$\text{Rt }=\;\left(\;\begin{array}{cc} \cos ɵ & -\text{sin}ɵ\\ \text{sin}ɵ & \text{cos}ɵ \end{array}\;\right)$$

The value of ɵ helps to rotate the image around the center pixel. In this experimental scenario, a range of 0 to 150 was used as a value of angle ɵ.

### Flips

As the name suggests, it includes both vertical and horizontal reflections of pixels. It is a well-known technique applied in the agricultural field for data augmentation (Pandian et al., 2019). It captured the unique properties of agricultural images. In the present study, we successfully implemented both vertical and horizontal flips for augmentation.

### Noise injection

It is the unwanted information embedded with the original signal that has produced a new image. In this scenario, Gaussian noise, spackle noise, random noise, and salt and pepper noise are the most common variants of noise. We used Gaussian noise for augmentation to produce a new training dataset for our experiment.

### Mixup

This technique consists of taking two images as input, merging them at a certain weight (*λ*), and generating a new image *X*’. Although this technique seems unusual, it demonstrates superior results with well-known datasets (Harris et al., 2020; Li et al., 2023).


.(4) $$X'=\lambda X_{i}+\left(1-\lambda \right)\;X_{j},\;\lambda \;\epsilon \text{ range}\left(0,1\right)$$

*X*’ is the newly generated image, *λ* is the weight factor, *X_i_* is the first image, and *X_j_* is the second image.

Data augmentation with image mixing is useful in different application areas. For this reason, we used mixing for data augmentation. In the present study, we used two different images of the same pet class with *λ* = 0.2 to generate a new image.

### Scaling

It introduced zoom-in/out transformation with shape variations depending on value magnitude. Here, we used scale in the transformation and cropped a specific portion of the images to the new augmented dataset for comparison.

### Principal component analysis

For the comparative analysis, we used principal component analysis for dimensionality reduction. It is a well-known synthetic data augmentation technique first used in AlexNet (Krizhevsky et al., 2012) and later (Geetharmani and Pandian, 2019; Tongcham et al., 2020) considered for plant leaf disease detection. In this experimental setup, we used a different number of components for dimensionality reduction. The value of component *k* is dependent on the variance. We used a level of 95% variance value for synthetic data generation.

### Enhancement

As per the name, the method is used for image enhancement. We used a histogram equalizer to make a new augmented dataset. In this experiment, we split the RGB image into different channels and perform histogram equalization to stretch out the intensity value of the image.

The authors perform different experimental scenarios to measure the efficiency of the proposed technique. In the baseline scenario, the original dataset was taken and evaluated with all three models. The experiment’s objective is to assess the system’s baseline performance. **Table 3** shows the impact of different augmentation methods and gives an idea to the readers of their relevance. This table shows the performance of different data augmentation methods described in terms of validation accuracy achieved by the machine vision model during the training period. The results achieved by the different updated models with the proposed augmentation pipeline significantly improve the validation accuracy.

**Table 3:** Comparative analysis of different benchmarking models through validation accuracy of different machine vision models.

Data augmentation sets	Augmentation approach	Model		
		VGG16	VGG19	ResNet50
**Baseline set 0**	No augmentation	71.123%	72.552%	74.852%
**Data augmentation 1**	Rotation	73.825%	74.026%	76.295%
**Data augmentation 2**	Vertical + horizontal flips	62.895%	75.501%	78.296%
**Data augmentation 3**	Contrast enhancement	71.742%	76.426%	71.973%
**Data augmentation 4**	Noise	75.051%	74.039%	69.126%
**Data augmentation 5 (** Tongcham et al., 2020 **)**	PCA	74.042%	72.693%	69.135%
**Data augmentation 6** (Li et al., 2023)	Mixup	76.124%	69.516%	66.761%
**Data augmentation 7**	Scale in/out	74.412%	65.512%	68.915%
**Data augmentation 8 (** Li et al., 2019 **)**	Rotation + scaling	74.035%	75.247%	75.508%
**Data augmentation 9** (Huang et al., 2022)	Rotation + flip + scaling	78.897%	74.717%	69.433%
**Data augmentation 10**	Proposed approach	**82.182%**	**76.713%**	74.122%

Bold values shows highest achieved results.

**Figure 5:**
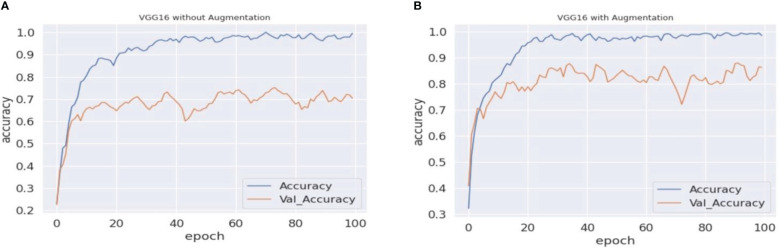
VGG16 accuracy comparison. **(A, B)** The graphical representation of validation and training accuracy results of the VGG16 architecture on augmentation and nonaugmentation datasets for comparison. During the training process with the baseline dataset, the training curve reaches a saturation point after approximately 40 epochs, while the validation curve shows oscillation in the overall training process. In **(B)**, on the other hand, the training curve reaches a saturation point after approximately 30 epochs, while the validation curve shows better results and less oscillation.

**Figure 6:**
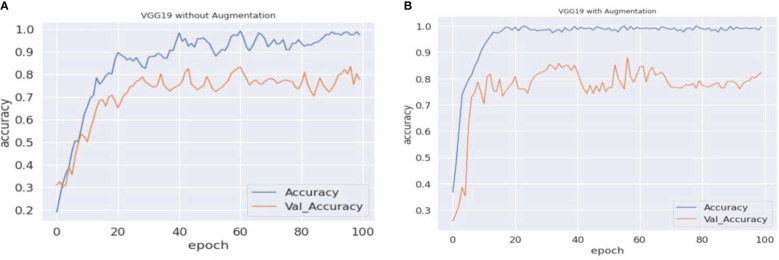
VGG19 accuracy comparisons. **(A, B)** The graphical representation of validation and training accuracy results of the VGG19 architecture on augmentation and nonaugmentation datasets for comparison. During the training process with the baseline dataset, the training curve reaches a saturation point after approximately 85 epochs, while the validation curve shows oscillation in the overall training process. In **(B)**, on the other hand, the training curve reaches a saturation point after approximately 80 epochs, while the validation curve regularly shows oscillation.

**Figure 7:**
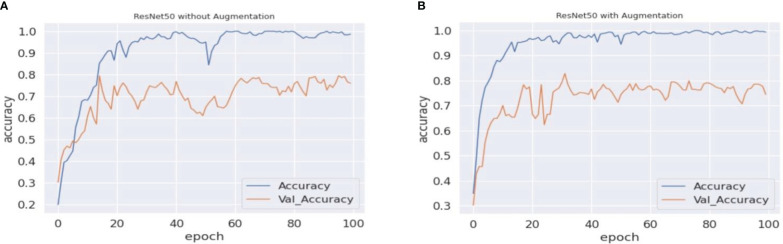
ResNet50 accuracy comparisons. **(A, B)** The graphical representation of validation and training accuracy results of the ResNet50 architecture on augmentation and nonaugmentation datasets for comparison. During the training process with the baseline dataset, the training curve reaches a saturation point after approximately 33 epochs, while the validation curve shows oscillation in the overall training process. In **(B)**, on the other hand, the training curve reaches a saturation point after approximately 30 epochs, while the validation curve regularly shows oscillation.

As previously stated in the last section, the experiment in this paper involves VGG16, VGG19, and ResNet50 with an updated fully connected head. The training of the model is performed over 100 epochs. **Table 3** represents the validation accuracy achieved by each experimental set. The validation accuracy represents the generalization capability of models on unseen data. During the training process of machine vision models, the validation accuracy is measured to test the model’s real-time problem-solving capabilities. In the baseline scenario (baseline dataset 0), models achieved 71.23%, 72.55%, and 74.85% validation accuracy, respectively. The validation accuracy on each experimental scenario using different augmentation datasets improves the model’s capabilities and helps in the maintenance of the overfitting problem. In the case of the proposed approach, the VGG16 and VGG19 models surpassed the other state-of-the-art validation accuracy and achieved 82.18% and 76.71% validation accuracy, respectively. In the present study, we also traversed different domains of data augmentation for agricultural images. Surprisingly, the synthetic data augmentation method such as mixing and PCA augmentation also help boost models’ performance over the traditional augmentation methods. The results showed that the validation accuracy achieved by the methods helped improve the performance of the VGG16 model. In addition, **Figures 5**–**7** represent a performance comparison of selected machine vision models on the baseline with the proposed experimental run.

**Figure 8:**
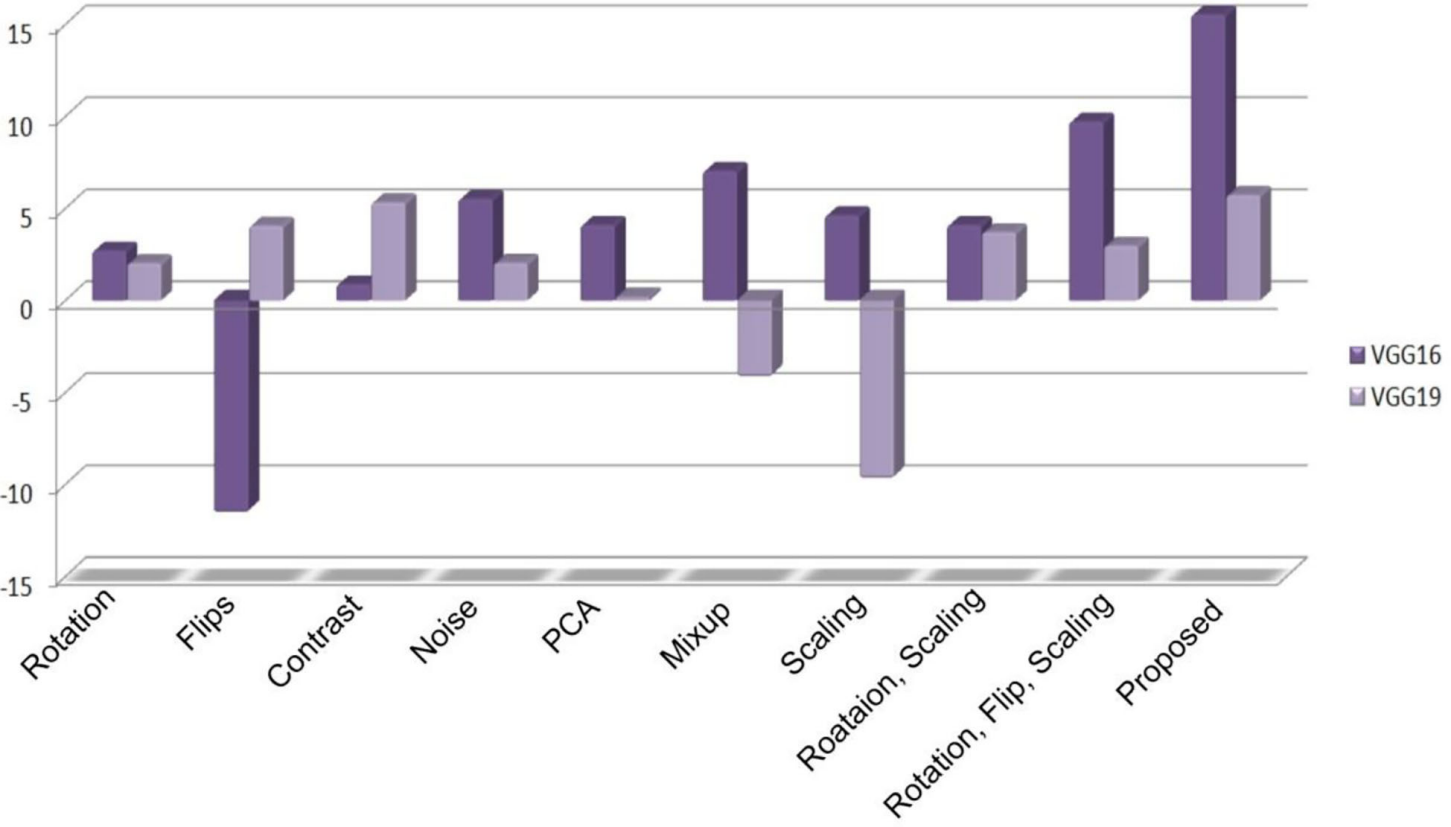
Average classification accuracy obtained by models.

Furthermore, following the evaluation of different machine vision models on the proposed data generation pipeline and different cumulative results, the authors provide a detailed assessment of various state-of-the-art models. The purpose of this assessment is to analyze the contribution made by selected state-of-the-art techniques in data generation. To make this objective clear, nine different datasets with help of selected state-of-the-art techniques were generated and have been processed as training datasets. The original image dataset was used as a validation dataset. The purpose of the selected scenario is to measure the contribution of each state-of-the-art’s contribution to the model’s baseline performance. In this regard, **Figure 8** demonstrates the percentage of contribution produced by the selected augmentation strategies in comparison to the validation accuracy. The resulting accuracy of the VGG16 and VGG19 models was almost 82.182% and 76.713%, respectively, with the data augmentation method. The performance analysis highlights that the application of the proposed data augmentation method contributes to an overall 15.54% improvement in VGG16 and 5.73% in VGG19. In addition, the negative responses are produced by VGG16 and VGG19. Overall, the assessments of possible outcomes indicate that the data augmentation strategies resulted in improvements in machine vision models. Furthermore, we traversed different domains of synthetic data augmentation methods for agricultural imaging, such as PCA and image mixing. Surprisingly, these methods also generated positive contributions over the machine vision models trained on different traditional data augmentation methods. The detailed analysis of the results reveals that the proposed data augmentation technique improved classification performance with a disproportional impact.

## Discussion

The authors explored a novel manipulation-based data augmentation technique. The proposed strategy yields positive results and strengthens the machine vision model for accurate insect prediction. Furthermore, the suggested strategy does not necessitate any specific hyperparameter and ensures a high degree of variation in the targeted dataset. Following the analysis, the results given in the preceding section reveal that the original dataset showed excessive fluctuations and poor performance. However, when the augmentation procedure is used, the prediction performance of the aforementioned class improves, as demonstrated by the augmented dataset. Similarly, the insect pest classification results reveal that the proposed fully connected head for ResNet50, VGG16, and VGG19 improved the results under the proposed data-augmented process. Although there are various studies included in this literature, employing data augmentation processes improved classification performance from 0.19% to 9.7%. To the best of our knowledge, similar strategies have not been published for insect pest classification. The suggested data augmentation procedure increased the robustness of the training model by using a cascaded strategy of multiple manipulation-based transformations of insect pest images.

The authors explored the domain of manipulation-based data augmentation for insect pest images. This technique may be viewed as a form of elastic transformation, which also brings to the discussion why the technique yields superior results. One probable explanation is that manipulation-based approaches preserve the actual feature information of images during the transformation. It can help increase the randomness in the image dataset and increase the model’s robustness. Rosa et al. (2022) ran an experiment on different geometric-based transformations on different datasets. This technique is a subpart of a manipulation-based technique and encourages robustness. However, after training the model with the augmented dataset, a tipping point occurs where the accuracy level shoots up. The asymptotic behavior of the accuracy along with each incremental step is also clearly noticeable. After a specific point, a computer’s effort to generate increasingly more synthetic data may not be worthwhile.

## Conclusion

The authors have studied the influence of the manipulation-based data augmentation technique for precision agriculture. The suggested technique expands the availability of the insect pest dataset of castor crops for classification. The proposed model enhances the VGG16 and VGG19 frameworks by extracting features from the traditional network and classifying features with the help of an updated, fully connected head. The evaluation of the proposed technique was achieved with help of 10 datasets and three deep learning frameworks. The results of different architectures reveal that the proposed manipulation-based data augmentation process improves accuracy by 15.54% and 5.73% for VGG16 and VGG19, respectively. After the investigation, it has been concluded that the data generation procedure of the proposed technique would inevitably improve model performance for castor insect pest prediction. The authors also noted that tasks related to deep learning face multiple challenges, and data unbalancing is one of them. The future study will involve a detailed investigation of several data augmentation subfields and an attempt to optimize these techniques using genetic algorithms.

## Data availability statement

The original contributions presented in the study are included in the article/supplementary material. Further inquiries can be directed to the corresponding authors.

## Author contributions

N, SG, RY, FB, PT these authors contributed equally to this work. All authors contributed to the article and approved the submitted version.
